# Psychotropic medication use during preanesthetic evaluation at a Brazilian university hospital: a retrospective cross-sectional analysis

**DOI:** 10.1016/j.bjane.2026.844783

**Published:** 2026-06-22

**Authors:** Matheus Salera Gordim, Vitor Yan Bezerra de Araújo, Danilo de Oliveira da Câmara Filho, Luzinelson Muniz da Silva Júnior, Emerson Arcoverde Nunes, Heleno Paiva Oliveira, Wallace Andrino da Silva

**Affiliations:** a Universidade Federal do Rio Grande do Norte (UFRN), Natal, Rio Grande do Norte, Brazil; b Universidade Federal do Rio Grande do Norte (UFRN), Hospital Universitário Onofre Lopes, Department of Surgery (Anesthesiology Residency Program), Natal, RN, Brazil; c Universidade Federal do Rio Grande do Norte (UFRN), Hospital Universitário Onofre Lopes, Department of Internal Medicine, Natal, RN, Brazil; d Universidade Federal do Rio Grande do Norte (UFRN), Hospital Universitário Onofre Lopes, Department of Surgery, Natal, RN, Brazil

Dear Editor,

The global rise in mental health disorders has been accompanied by increased use of psychotropic medications, which are now among the most frequently prescribed drug classes worldwide.^1^ In surgical patients, chronic psychotropic use is clinically relevant because these agents may interact with anesthetics through pharmacokinetic and pharmacodynamic mechanisms.^2^ Despite this, contemporary epidemiological data on psychotropic use among surgical patients remain limited.

We conducted a retrospective cross-sectional analysis using routinely collected electronic medical record data from the anesthesiology outpatient clinic of the Onofre Lopes University Hospital, Brazil. Adult patients (≥ 18 years) evaluated between January and December 2024 were consecutively included. Records without medication data were excluded. Duplicate records were filtered by retaining the most complete or most recent entry. Records with missing values for variables such as Body Mass Index (BMI) were retained for prevalence analyses, but excluded from statistical tests requiring those data.

Extracted variables included demographics, clinical characteristics, surgical specialty, and psychotropic medication use. Current psychotropic use ‒ encompassing both scheduled and as-needed use ‒ was determined by patient self-report during preanesthetic consultation and documented in the medical record. Without prior prespecification, reported agents were retrospectively categorized into therapeutic classes (antidepressants, anxiolytics/hypnotics, antipsychotics, mood stabilizers, and psychostimulants). Polypharmacy was defined as the concurrent use of two or more psychotropic agents.

Comparisons involved Chi-Square tests for categorical variables and Student's *t*-test or Mann-Whitney *U*-tests for continuous variables (age and BMI), with normality assessed via the Shapiro-Wilk test. Logistic regression estimated Odds Ratios (OR) with 95% Confidence Intervals. Models included age, sex, and BMI category a priori; additional variables were excluded due to data heterogeneity and to preserve parsimony.

A total of 1,038 patients were included in the final analysis, of whom 23.0% (n = 239) were using at least one psychotropic medication (Table 1). This prevalence is higher than rates reported in Brazilian community-based studies, which generally range from 10% to 18%, suggesting that surgical patients may represent a subgroup with greater psychiatric burden and healthcare utilization.

**Table 1:** Demographic and clinical characteristics of patients.

Variable	Psychotropic use (n = 239) n (%) or mean ± SD	No psychotropic use (n = 799) n (%) or mean ± SD	p-value
**Sex**, n (%)			< 0.001ᵇ
Male	62 (14.12)	377 (85.88)	
Female	177 (29.55)	422 (70.45)	
**Age** (years), mean ± SD	49.54 ± 14.42	50.99 ± 15.44	0.182ᵃ
**BMI** (kg.m^-2^), mean ± SD	34.49 ± 10.48	32.13 ± 9.89	0.005ᵃ
**BMI category**, n (%)			0.066ᵇ
Underweight (BMI < 18.5 kg.m^-2^)	1 (7.14)	13 (92.86)	
Normal weight (BMI 18.5–24.9 kg.m^-2^)	26 (19.25)	109 (80.74)	
Overweight (BMI 25.0–29.9 kg.m^-2^)	40 (19.32)	167 (80.67)	
Obesity class I (BMI 30–34.9 kg.m^-2^)	29 (24.57)	89 (75.42)	
Obesity class II (BMI 35–39.9 kg.m^-2^)	25 (30.48)	57 (69.51)	
Obesity class III (BMI ≥ 40.0 kg.m^-2^)	43 (29.05)	105 (70.94)	
**Surgical specialty**, n (%)			< 0.001ᵇ
General Surgery	57 (19.86)	230 (80.14)	
Digestive Tract Surgery	84 (32.18)	177 (67.81)	
Urology	20 (12.26)	143 (87.73)	
Vascular Surgery	22 (19.81)	89 (80.18)	
Plastic Surgery	21 (29.57)	50 (70.42)	
Thoracic Surgery	11 (36.67)	19 (63.33)	
Other Specialties	24 (20.87)	91 (79.13)	

SD, Standard Deviation; BMI, Body Mass Index; ᵃ *t*-test, ᵇ Chi-Square test.

Note: The total 'n' for BMI variables (n = 704) differs from the total sample due to missing data in medical records. For categorical variables, percentages indicate the proportion within each category/row, not column percentages.

Psychotropic medication use was significantly more frequent among women, affecting 29.55% of women compared with 14.12% of men (p < 0.001). In a multivariable logistic regression adjusted for age and BMI category, female sex remained independently associated with a higher likelihood of psychotropic medication use (OR = 2.74; 95% CI 1.80‒4.15; p < 0.001) (Table 2), while age was not significantly associated with the outcome (p = 0.440). Due to missing BMI data, this multivariable model included 704 patients. Biological factors, including hormonal fluctuations influencing serotonergic and GABAergic neurotransmission, along with psychosocial determinants such as cumulative stress and social role burden, may contribute to this disparity.^3^

**Table 2:** Logistic regression analysis of factors associated with psychotropic drug use.

Variable	Category/Comparison	OR	95% CI	p-value
**Sex (unadjusted)**	Female vs. Male	2.52	1.83 ‒ 3.49	**< 0.001**
**Age**	Per year increase	0.99	0.98 ‒ 1.01	0.44
**Sex (adjusted)**	Female vs. Male	2.74	1.80 ‒ 4.15	**< 0.001**
**BMI category**^a^	Underweight	0.30	0.03 ‒ 2.52	0.27
	Overweight	0.95	0.54 ‒ 1.66	0.85
	Obesity class I	1.05	0.56 ‒ 1.95	0.88
	Obesity class II	1.29	0.67 ‒ 2.51	0.44
	Obesity class III	1.17	0.65 ‒ 2.10	0.59

OR, Odds Ratio; CI, Confidence Interval.

a Reference category: normal weight (BMI 18.5–24.9 kg.m^-2^). The multivariable model included age, sex, and BMI category and was fitted in 704 patients with complete BMI data. The adjusted OR for sex is adjusted for age and BMI category; BMI category estimates are adjusted for age and sex.

Mean BMI was higher among psychotropic users (34.49 ± 10.48 kg.m^-2^) than among non-users (32.13 ± 9.89 kg.m^-2^; p = 0.005). Although no independent association between categorical BMI and psychotropic use remained after adjustment, this finding is consistent with literature showing a bidirectional relationship between obesity and psychiatric disorders.^4^ From a perioperative perspective, obesity combined with psychotropic use may increase the risk of difficult airway management, hypoventilation, and obstructive sleep apnea. Psychotropic medication use also varied across surgical specialties, with higher proportions observed in digestive tract, plastic, and thoracic surgeries.

The pharmacological profile revealed that antidepressants were the most frequently prescribed therapeutic class (71.97%), followed by anxiolytics/hypnotics (30.13%), mood stabilizers (24.69%), and antipsychotics (19.67%) (Table 3). Clonazepam, sertraline, and fluoxetine were the most commonly prescribed individual medications.

**Table 3:** Distribution of psychotropic medications by therapeutic class, individual agents and polypharmacy.

Therapeutic class	n	%
Antidepressants	172	71.97
Anxiolytics/Hypnotics	72	30.13
Mood stabilizers	59	24.69
Antipsychotics	47	19.67
Psychostimulants	3	1.26
**Psychotropic medication**	**n**	**%**
Clonazepam	54	22.59
Sertraline	46	19.25
Fluoxetine	43	17.99
Amitriptyline	31	12.97
Escitalopram	28	11.72
Quetiapine	26	10.88
Pregabalin	22	9.21
Topiramate	13	5.44
Risperidone	12	5.02
Sodium valproate	10	4.18
Desvenlafaxine	10	4.18
**Number of psychotropic medications**	**n**	**%**
One	139	58.16
Two	61	25.52
Three or more	39	16.32

Note: Percentages were calculated among psychotropic medication users (n = 239). Therapeutic classes and individual medications are not mutually exclusive.

Psychotropic polypharmacy was observed in 41.84% of users, representing a relevant point for preoperative medication review and individualized anesthetic planning. The concurrent use of multiple psychotropic medications may increase the potential for pharmacokinetic and pharmacodynamic interactions in the perioperative setting, including cytochrome P450 modulation, GABA-A receptor adaptation, and QT interval prolongation.^2^^,^^5^ While our study was fundamentally descriptive and did not evaluate clinical outcomes, these mechanisms may contribute to altered anesthetic requirements and autonomic instability.

Current consensus favors individualized perioperative management rather than routine discontinuation of psychotropic medications. Abrupt withdrawal of antidepressants and anxiolytics may precipitate psychiatric destabilization, whereas medications such as monoamine oxidase inhibitors and lithium require specific perioperative strategies because of their interaction profiles.^2^

This study has limitations inherent to its retrospective and cross-sectional design, including reliance on electronic medical records, absence of dosage and treatment duration data, and inability to correlate psychotropic exposure with perioperative outcomes. Nevertheless, the large sample and standardized extraction methods provide a relevant epidemiological overview of psychotropic medication use in a tertiary surgical population.

In summary, nearly one in four patients presenting for preanesthetic evaluation in our tertiary university hospital was using a psychotropic medication, with frequent polypharmacy and a higher prevalence among women. Antidepressants and benzodiazepines predominated. These findings reinforce the importance of psychotropic medication reconciliation during preanesthetic assessment. Future prospective studies should evaluate the relationship between psychotropic exposure patterns and postoperative outcomes to better guide anesthetic planning.

## AI assistance disclosure

Claude AI was used exclusively to assist with language revision and formatting of the English text. No AI system was involved in the study design, data collection, data analysis, interpretation of results, or manuscript content generation. The authors take full responsibility for the content of the publication.

## Authorship Criteria (ICMJE)

All authors meet the four International Committee of Medical Journal Editors (ICMJE) criteria for authorship: (1) Substantial contributions to the conception or design of the work, or the acquisition, analysis, or interpretation of data; (2) Drafting the work or revising it critically for important intellectual content; (3) Final approval of the version to be published; and (4) Agreement to be accountable for all aspects of the work.

## Ethics approval

The study was approved by the local Research Ethics Committee in accordance with national regulations for research involving human subjects (CAAE: 91167425.10000.5292).

## Authors’ contributions

Matheus Salera Gordim, Vitor Yan Bezerra de Araújo, Luzinelson Muniz da Silva Júnior and Danilo de Oliveira da Câmara Filho contributed to data collection, data analysis, and drafting of the manuscript. Heleno Paiva Oliveira, Emerson Arcoverde Nunes and Wallace Andrino da Silva contributed to study supervision, data analysis, critical revision of the manuscript for important intellectual content, and approval of the final version to be published. All authors approved the final version of the manuscript and agree to be accountable for all aspects of the work.

## Funding

The authors received no specific funding for this work.

## Conflicts of interest

The authors declare no conflicts of interest.

## Data Availability

The datasets generated and/or analyzed during the current study are available from the corresponding author upon reasonable request.
